# Rootstock effects on leaf function and isotope composition in apple occurred on both scion grafted and ungrafted rootstocks under hydroponic conditions

**DOI:** 10.3389/fpls.2023.1274195

**Published:** 2023-12-13

**Authors:** Erica Casagrande Biasuz, Lee Kalcsits

**Affiliations:** ^1^ Tree Fruit Research and Extension Center, Washington State University, Wenatchee, WA, United States; ^2^ Department of Horticulture, Washington State University, Pullman, WA, United States

**Keywords:** Malus domestica, carbon isotope composition, oxygen isotope composition, nitrogen isotope composition, leaf gas exchange, scion-rootstock interactions

## Abstract

Rootstocks are used in modern apple production to increase productivity, abiotic and biotic stress tolerance, and fruit quality. While dwarfing for apple rootstocks has been well characterized, the physiological mechanisms controlling dwarfing have not. Previous research has reported rootstock effects on scion water relations. Root architecture and variability in soil moisture across rooting depths can also contribute to these differences among rootstocks in the field. To exclude these effects on rootstock behavior, scions were grafted onto four different rootstocks with varying effects on scion vigor (B.9, M.9, G.41 and G.890). Non-grafted rootstocks were also grown to examine whether the effects of rootstock occurred independently from scion grafting. Plants were grown in a greenhouse under near steady-state hydroponic conditions. Carbon (δ^13^C), oxygen (δ^18^O) and nitrogen (δ^15^N) isotope composition were evaluated and relationships with carbon assimilation, water relations, and shoot growth were tested. Rootstocks affected scion shoot growth, aligning with known levels of vigor for these four rootstocks, and were consistent between the two scion cultivars. Furthermore, changes in water relations influenced by rootstock genotype significantly affected leaf, stem, and root δ^13^C, δ^18^O, and δ^15^N. Lower δ^13^C and δ^18^O were inconsistently associated with rootstock genotypes with higher vigor in leaves, stems, and roots. G.41 had lower δ^15^N in roots, stems, and leaves in both grafted and ungrafted trees. The effects of rootstock on aboveground water relations were also similar for leaves of ungrafted rootstocks. This study provides further evidence that dwarfing for apple rootstocks is linked with physiological limitations to water delivery to the developing scion.

## Introduction

The use of composite plants consisting of a rootstock and scion is necessary for modern tree fruit production. Rootstocks impart beneficial traits such as resistance or tolerance to abiotic or biotic stresses (Garner, 2013), yield efficiency, and fruit quality (Palmer and Wertheim, 1980; Sansavini et al., 1980; Mika et al., 2000; Reig et al., 2018; Reig et al., 2020). Dwarfing rootstocks can allocate more energy to fruit production increasing both fruit quality and yield (Melnyk and Meyerowitz, 2015). Dwarfing rootstocks limit scion vegetative growth (Cohen and Naor, 2002; Tworkoski and Miller, 2007; Hayat et al., 2020) and this effect has been extensively described (Reviewed in Marini and Fazio, 2018). Still, the mechanisms controlling rootstock effects on vigor are poorly understood and may be multifaceted (Beakbane and Thompson, 1940; Webster, 2002; Gregory et al., 2013). Rootstocks may influence hormone transport limiting scion growth (Tworkoski and Fazio, 2016; Foster et al., 2017; Lordan et al., 2017; Adams et al., 2018). In some cases, the graft union itself may also limit xylem connectivity that may impede water and mineral movement to the scion (Atkinson et al., 2003; Tworkoski and Fazio, 2008; Bauerle et al., 2011). In other examples, root architecture and distribution can also affect nutrient and water uptake which could also affect scion growth and development (Hayat et al., 2020). Lower hydraulic conductivity has been reported for dwarfing rootstocks compared to more vigorous rootstocks (Atkinson and Else, 2001; Atkinson et al., 2003). These effects have also been reported in olives (Nardini et al., 2006), grapes (Shtein et al., 2017), and citrus (Martínez-Alcántara et al., 2013). The common effects among these proposed mechanisms are limitations to water and solute movement through the soil-plant-atmosphere continuum (Atkinson and Else, 2001; Xu and Ediger, 2021; Xu et al., 2021).

Water relations are regulated with direct transport of water from the soil to the leaves through the xylem (Olien and Lakso, 1986). Resistance has been reported to increase as the dwarfing effect increases (Atkinson et al., 2003; Tworkoski and Fazio, 2015). Furthermore, Olien and Lakso (1984) suggested that lower water potential for dwarfing rootstocks is not caused by resistance to water transport over the whole tree but exclusively by the rootstock (Olien and Lakso, 1984). Dwarfing rootstocks generally have less leaf area compared with vigorous rootstocks. Moreover, dwarfing effect is associated with scion precosity and, thus, earlier partitioning of photosynthates to fruit production occurs (Tworkoski and Fazio, 2015). However, dwarfing traits can also be observed in non-fruiting trees. The effect of dwarfing rootstocks is hypothesized to be associated with water relations, but the location of these limitations remains unclear.

Stable isotope composition can be useful for evaluating the effect of rootstock on scion growth and is an integrated measure of water-use efficiency and water relations. During carbon assimilation, ^12^C is discriminated against over the heavier isotope ^13^C at both the diffusion step into the mesophyll and during fixation by Rubisco, so δ^13^C of fixed carbon becomes depleted relative to atmospheric carbon dioxide (Farquhar et al., 1989; Wallace et al., 2013). Discrimination during carbon fixation is largely regulated by stomatal conductance. More conservative plants limit the inflow of CO_2_ into the leaf and thus, reduce the plant discrimination rates against ^13^C. Stomatal conductance is directly affected by plant water status, thus δ¹³C serves as a proxy method for water relations studies in plants (Farquhar et al., 1989; Wallace et al., 2013). Oxygen isotope composition can be used to separate physiological processes like leaf stomatal conductance and transpiration rate, especially when integrated with δ^13^C (Barbour, 2007). Since δ^13^C provides an integrative record of supply and demand for CO_2_, combining δ^13^C and δ^18^O could enable the separation of stomatal and photosynthetic effects from the rootstock (Barbour, 2007) and as consequence, acquire a stronger understanding of water use efficiency within the plant (Cernusak, 2020). Nitrogen isotope composition can be an indicator of N assimilation processes (Evans, 2001; Kalcsits et al., 2014) and the linkage between these three isotope measurements has been established for agricultural crops (Yousfi et al., 2013). These isotopes are often integrators of environmental conditions in the soil. However, soil-based heterogeneity can often mask rootstock-driven differences in isotope composition which may help shed light on the mechanisms driving tolerance to abiotic stress and environmental plasticity.

Being the interface between soil water supplies and photosynthetic tissues above-ground, rootstocks clearly have a significant role in improving tolerance to abiotic stresses such as water limitations under a changing climate. The objective for this study was to assess how rootstocks affect plant water relations and stable isotope composition when grown in a near steady-state hydroponic conditions. These approaches will help discern between architectural and soil-based interactions that can occur in the field from rootstocks and physiological limitations occurring in the root or graft union. We hypothesized that dwarfing rootstocks limit water uptake to aboveground tissue thus reducing transpiration rate, stomatal conductance, and photosynthesis and that rootstock effects are present when a scion is grafted to the rootstock or are left ungrafted.

## Materials and methods

### Greenhouse hydroponic system and growth conditions

**Figure 1:**
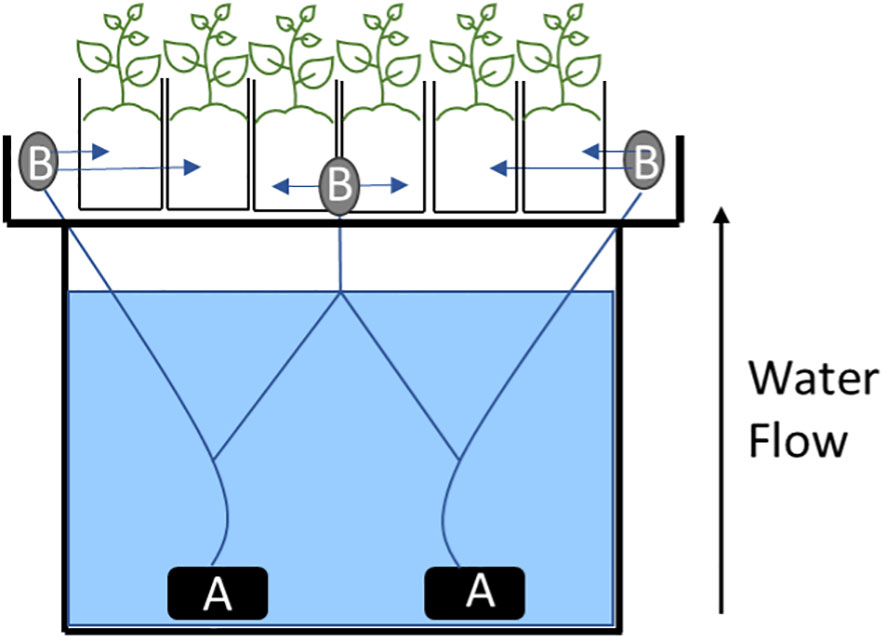
Schematic for the hydroponic system. **(A)** two water pumps used to propel water up to the plants. **(B)** three 8-Port NPT Irrigation Manifold used to distribute the water equally into each one of the 24 pots.

The hydroponic system was comprised of six custom-built 455 L holding tanks placed in temperature-controlled greenhouse at Washington State University – Tree Fruit Research and Extension Center (WSU-TFREC), Wenatchee, WA (47.438127, -120.346656) with temperatures maintained between 20-23°C (**Table 1**). The wooden containers were lined with black rubber pond liners (Total Pond, West Palm Beach, Florida) and covered by a 75 x 75 cm tray (Botanicare-Agron, Aurora, CO) containing 24.5 L pots filled with perlite (PVP Industries, Orwell, OH). The hydroponics solution was a modified 1/10th Hoagland’s nutrient solution with concentrations of the following mineral salts: 60 mg L-1 of KNO_3_, 50 mg L^-1^ of MgSO_4_*7H_2_O, 95 mg L^-1^ of CaNO_3_*4H_2_O, 15 mg L^-1^ of (NH4)_3_PO_4_. For micronutrients, a stock solution was prepared at 1000x the target concentrations and then 1 mL L-1 of stock solution was added to each container with final concentrations of: 500 μg L^-1^ of KCl, 250 μg L^-1^ of H_3_Bo_3_, 50 μg L-1 of MnCl_2_, 20 μg L^-1^ of ZnSO_4_*7H_2_O, 5 μg L^-1^ of CuSO_4_*5H_2_O, 1 μg L^-1^ of H_2_MoO_4_ (85%), 500 g L^-1^ of FeSO_4_*7H_2_O and 300 μg L^-1^ of NaEDTA. pH was maintained between 6.5-7.0. The solution was circulated from the holding tank to the hydroponic tray using two submersible water pumps (3500L H-1, 60W) (Vivosun, Ontario, CA) per container through three 8-Port NPT Irrigation Manifold (Orbit, North Salt Lake, UT) placed on the front and sides of each tray (**Figure 1**). An air pump (EcoPlus 13500 LPH, Sunlight Supply Inc., Vancouver, WA) was connected to four air stone (Active Aqua ASCM Medium Air Stones, Hydrofarm Inc, Petaluma, Ca) which provided aeration at all times. Solution constantly circulated from the holding tanks to each pot during the experiment. Weather conditions such as air temperature and relative humidity (RH) were collected using an ATMOS 14 temperature & humidity sensor (Decagon Devices, Pullman, WA).

**Table 1:** Average greenhouse relative humidity and air temperature during May to August of 2019 during the experiment.

	Relative Humidity	Air Temperature
	(%)	(°C)
May	73.03	20.86
June	70.93	19.84
July	74.03	20.87
August	77.48	21.87

### Plant material and experimental design

**Figure 2:**
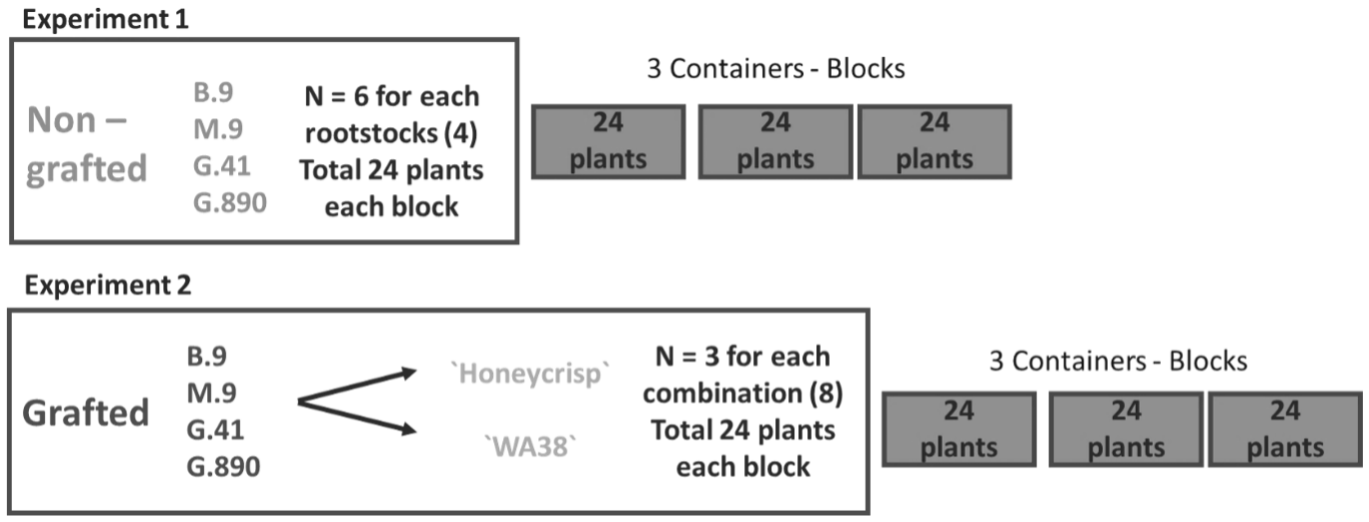
Experimental design for two greenhouse experiments. Experiment 1: Non-grafted plants experiment with six replicates for each rootstock placed individually in 24 pots for each experimental block. Experiment 2: grafted plants experiment with three replicates of each rootstock-scion combination placed individually in 24 pots for each the three experimental blocks.

Budagovsky 9 (B.9), Geneva 41 (G.41), Geneva 890 (G.890) and Malling M.9-T337 (M.9) rootstocks were selected to provide a range of known vigor levels among rootstocks. The accepted order in vigor from highest to lowest is; G.890>G.41>M.9-T337>B.9. Prior to grafting, dormant rootstock cuttings were stored for two months at 2°C in dark room. Budwood from two contrasting scion cultivars, ‘Honeycrisp’^®^ and `WA 38`^®^, was collected from Sunrise orchard (WSU-TFREC Sunrise Orchard, Rock Island, WA (47.311574, -120.067855) in February and placed in dark room until the time of the grafting. The two cultivars were grafted onto the rootstocks using a cleft graft. Nine composite plants were created for each combination of cultivar and rootstock. Eighteen other rootstock clones for each rootstock remained ungrafted. Three weeks after grafting, 72 ungrafted rootstocks (Experiment 1) and 72 grafted plants (experiment 2), were transferred to the greenhouse (**Figure 2**). The trees were placed into containers in a randomized complete block design (RCBD) with three containers as separate experimental blocks. Measurements were made after trees were grown for 60 days under hydroponic conditions.

### Tree measurements

Gas exchange was measured on one mature, fully expanded leaf from each of the trees to estimate CO_2_ assimilation, stomatal conductance, and intrinsic water use efficiency (iWUE as µmol CO_2_ mol H_2_O^-1^) (Equation 1), estimated as the ratio of net photosynthetic CO_2_ assimilation (A) to stomatal conductance (g_s_) using a Licor-6400XT (Li-Cor, Inc., Lincoln, NE, USA). Air flow was constant at 400 µmol s−1, reference CO2 concentration was set at 400 ppm, leaf temperature at 25°C, and photosynthetic photon flux density inside the chamber was set to 1,500 μmol m^−2^ s^−1^. After placing the leaf in the gas exchange chamber, the leaf was allowed to equilibrate until reference and sample values stabilized. Measurements were made every two weeks between 10:00 and 12:00 for two months on cloud-free days. Means were calculated for all sampling times during the season.

Equation 1


$$iWUE=\left(\frac{\text{Net Photosynthetic Rate}}{\text{Stomatal Conductance}}\right)$$

Stem water potential was measured on one fully mature expanded leaf selected from the bottom of each tree by placing individual leaves in a silver reflective bag for at least 90 minutes. At solar noon, stem water potential was measured using a Scholander System Pressure Chamber Instrument (PMS Instrument Co., Albany, OR, USA). Shoot length was measured from the bud scar of the only shoot allowed to develop to the apical meristem for each plant.

### Isotope analysis

Two mature leaves, vegetative stems (10 cm), and 1/3 of each root system were sampled for isotope composition. Samples were placed in paper bags and brought to the laboratory and dried. Dry leaves, stems and roots were well-mixed, and 2 g subsamples were ground to a fine powder using a VWR Homogenizer (VWR, Radnor, PA).

#### Oxygen isotope composition (δ18O) in biomass

0.6-0.8 mg samples for leaves and 1 mg samples for stems and roots was weighed using a high-precision analytical balance (XSE105 DualRange, Metler Toledo, Greifensee, Switzerland) into into 4x6 mm silver capsules (Costech Analystycal Technologies, Inc., Valencia, CA, USA). Prepared capsules were shipped for analysis to the Stable Isotope Core Laboratory at Washington State University. The samples were analyzed with continuous-flow pyrolysis using TC/EA interfaced with an IRMS (Delta Plus, ThermoFinnigan, Bremen, Germany) through a continuous flow device (Conflo-III, ThermoFinnigan,Bremen, Germany). Oxygen isotope ratios of each sample were then determined, and the values reported in “delta” notation as δ values in parts per thousand (‰):

Equation 2


$$\delta^{18}O=\left(\frac{\left(\frac{{\;}^{18}O}{{\;}^{16}O}\right)\;sample}{\left(\frac{{\;}^{18}O}{{\;}^{16}O}\right)standard}\;-1\right)\;X\;1000\left(‰\right)$$

δ18O was calculated as the ratio of the heavy isotope (18O) over the light oxygen isotope (16O) in the samples divided by the heavy isotope (18O) over the light oxygen isotope (16O) of the standard. The δ18O was compared with the standard Vienna-Standard Mean Ocean Water (VSMOW), the international standard commonly used for oxygen isotope analysis.

#### Carbon(δ13C) and nitrogen (δ15N) isotope composition in biomass

3 mg samples for leaves and 4 mg samples for stems and roots were weighed using a high-precision analytical balance (XSE105 DualRange, Metler Toledo, Greifensee, Switzerland) into 5mm x 9mm tin capsules (Costech Analystycal Technologies, Inc., Valencia, CA, USA). Prepared capsules were shipped for analysis to the Stable Isotope Core Laboratory at Washington State University. Samples were analyzed using an elemental analyzer (ECS 4010, Costech Analytical, Valencia, CA, USA) coupled with a continuous flow isotope ratio mass spectrometer (Delta PlusXP Thermofinnigan, Bremen). Carbon and nitrogen isotope ratios were determined using the standard Vienna PeeDee belemnite (VPDB) and the values reported in “delta” notations as δ values in permil (‰) as described in Equation 3 and 4 respectively:

Equation 3


$$\delta^{13}C=\left(\frac{\left(\frac{{\;}^{13}C}{{\;}^{12}C}\right)\;sample}{\left(\frac{{\;}^{13}C}{{\;}^{12}C}\right)standard}\;-1\right)\;X\;1000\left(‰\right)$$

Equation 4


$$\delta^{15}N=\left(\frac{\left(\frac{{\;}^{15}N}{{\;}^{14}N}\right)\;sample}{\left(\frac{15^{\;}N}{14^{\;}N}\right)standard}\;-1\right)\;X\;1000\left(‰\right)$$

### Statistical analysis

The experiment with rootstocks and scions was a randomized complete block design (RCBD) containing rootstocks (N=4) and scion (N=2) as factors with three blocks and three replicates of each rootstock-scion combination for each block. The experiment with non-grafted plants were also arranged as a RCBD with only rootstock (N=4) as a factor with three blocks and six replicates for each rootstock. Grafted and non-grafted plants were analyzed as separate experiments. Data were analyzed for effects of rootstocks and scions using two-way analysis of variance (ANOVA) on SAS 9.4 PROC GLM software (SAS Campus Drive Cary, NC, USA) for shoot length, net carbon assimilation, stomatal conductance, transpiration rate, midday stem water potential and δ13C, δ18O and δ15N. All treatment means were separated using Tukey`s means separation test with a confidence limit of 95%. Although cultivar was a factor in this statistical model, there were no interactions among cultivar and rootstock and, therefore, to focus on rootstock relations, scion cultivar effects were pooled and presented together. Linear regression was used to explore the relationships between variables and figures were prepared using OriginPro 2021 Data Analysis and Graphing Software (OriginLab Corporation, MA, USA).

## Results

### Water relations, gas exchange, and N content

**Figure 3:**
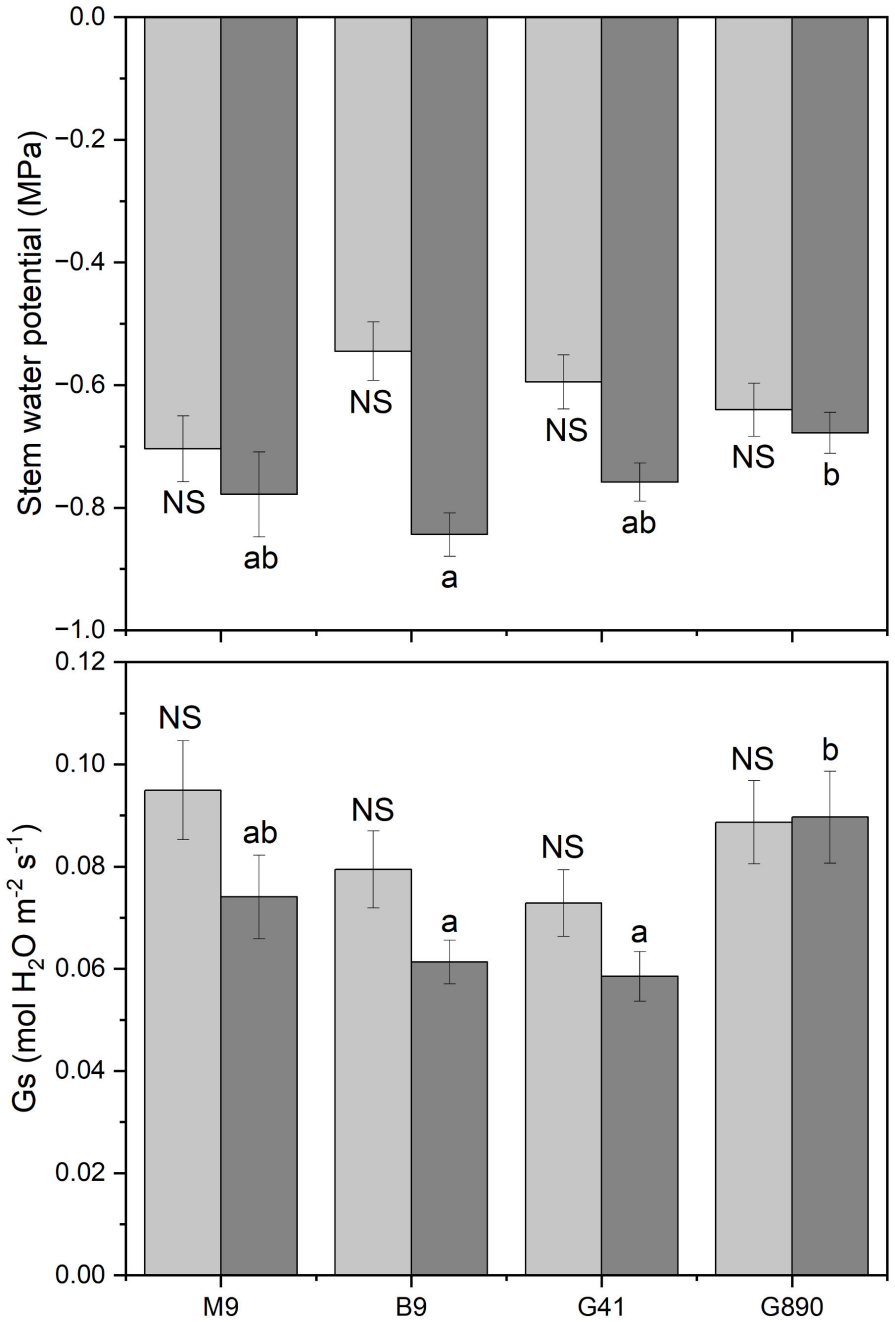
Stem water potential and stomatal conductance for non-grafted trees (light grey) and grafted trees (dark grey) for B.9, M.9, G.41 and G.890 rootstocks measured. Error bars denote standard error (N=3). Large and small letters indicate significance differences among means for ungrafted and grafted rootstocks, respectively, determined using a Tukey`s HSD mean separation test (α=0.05). ns indicates that the means are not significantly different.

**Figure 4:**
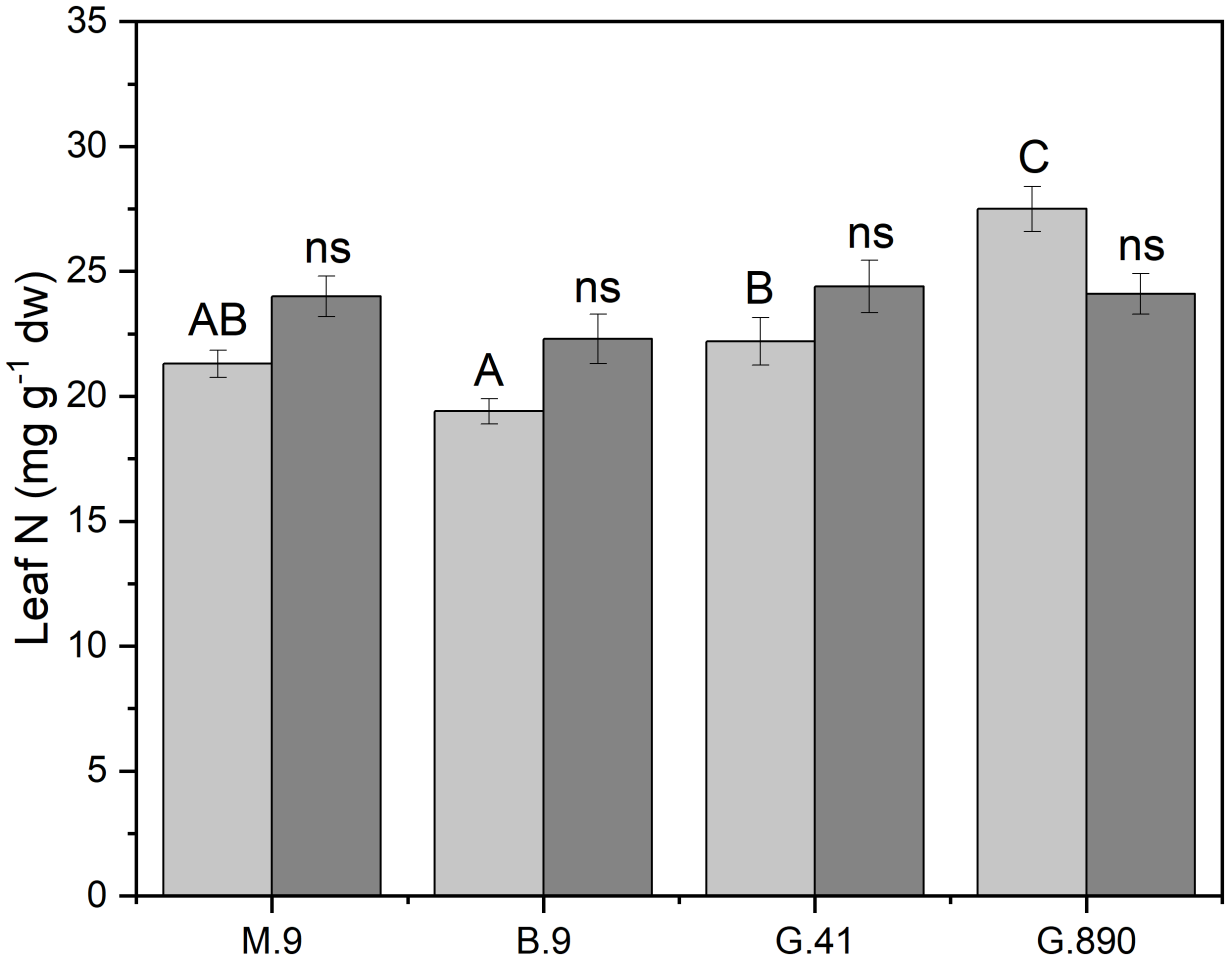
Leaf nitrogen content (mg g^-1^ dw) non-grafted trees (light grey) and grafted trees (dark grey) for B.9, M.9, G.41 and G.890 rootstocks measured. Error bars denote standard error (N=3). Large and small letters indicate significance differences among means for ungrafted and grafted rootstocks, respectively, determined using a Tukey`s HSD mean separation test (α=0.05). ns indicates that the means are not significantly different.

Midday stem water potential (Ψ_m_) significantly varied among rootstocks even under hydroponic conditions. For grafted plants, Ψ_m_ ranged from -0.68 MPa for G.890 to -0.84 MPa for B.9 (P< 0.05) (**Figure 3**; **Table 2**). M.9, G.41 and G.890 were not significantly different from one another. Similar patterns were also observed for stomatal conductance between G.890 and B.9. G.890 had higher stomatal conductance compared to B.9 (P<0.001) (**Figure 3**). For non-grafted trees, differences among rootstocks in stem water potential and stomatal conductance were smaller than for grafted trees. For ungrafted trees, M.9 had lower Ψ_m_ compared to B.9 (P = 0.05) (**Figure 3**). Moreover, M.9 had higher stomatal conductance than G.41 (P< 0.05) (**Figure 3**). Nitrogen content was highest for G.890 and lowest for B.9 with M.9 and G.41 with moderate N content when ungrafted (**Figure 4**). However, grafting dampened the effect of rootstock on leaf N content. There were no differences among rootstocks when they had a scion grafted onto them (P>0.05).

**Table 2:** Mean stem water potential (MPa), CO_2_ assimilation (µmol CO_2_ m^-2^ s^-1^), Stomatal conductance (mol H_2_O m^-2^ s^-1^) and Intrinsic Water use efficiency (iWUE; µmol CO_2_ mol H_2_O^-1^) ( ± SEM; N=6) for apple trees on M.9, B.9, G.41 and G.890 rootstocks.

	Stem water potential (MPa)	Net CO_2_ assimilation (µmol CO_2_ m^-2^ s^-1^)		Stomatal conductance (mol H_2_O m^-2^ s^-1^)	iWUE (µmol CO_2_ mol H_2_O^-1^)
Ungrafted plants					
M.9	-0.70 ± 0.05 a	4.91 ± 0.29 ns		0.095 ± 0.009 a	61.2 ± 7.96 ns
B.9	-0.55 ± 0.05 b	4.63 ± 0.31 ns		0.079 ± 0.007 ab	58.2 ± 4.47 ns
G.41	-0.59 ± 0.04 ab	4.66 ± 0.27 ns		0.073 ± 0.006 b	58.1 ± 3.36 ns
G.890	-0.64 ± 0.04 ab	5.17 ± 0.27 ns		0.088 ± 0.008 ab	56.7 ± 5.23 ns
Grafted plants					
M.9	-0.78 ± 0.07 ab	3.58 ± 0.16 ns		0.074 ± 0.008 ab	87.9 ± 24.38 ns
B.9	-0.84 ± 0.4 a	3.60 ± 0.47 ns		0.061 ± 0.004 a	72.7 ± 8.37 ns
G.41	-0.76 ± 0.03 ab	3.94 ± 0.29 ns		0.058 ± 0.004 a	86.9 ± 11.68 ns
G.890	-0.68 ± 0.03 b	4.32 ± 0.40 ns		0.089 ± 0.009 b	77.1 ± 10.17 ns

Different letters indicate significance differences among means determined using a Tukey`s mean separation test (α=0.05). ns indicates that the means are not significantly different.

### Tree growth and leaf, stem and root δ^13^C, δ^18^O, and δ^15^N

**Figure 5:**
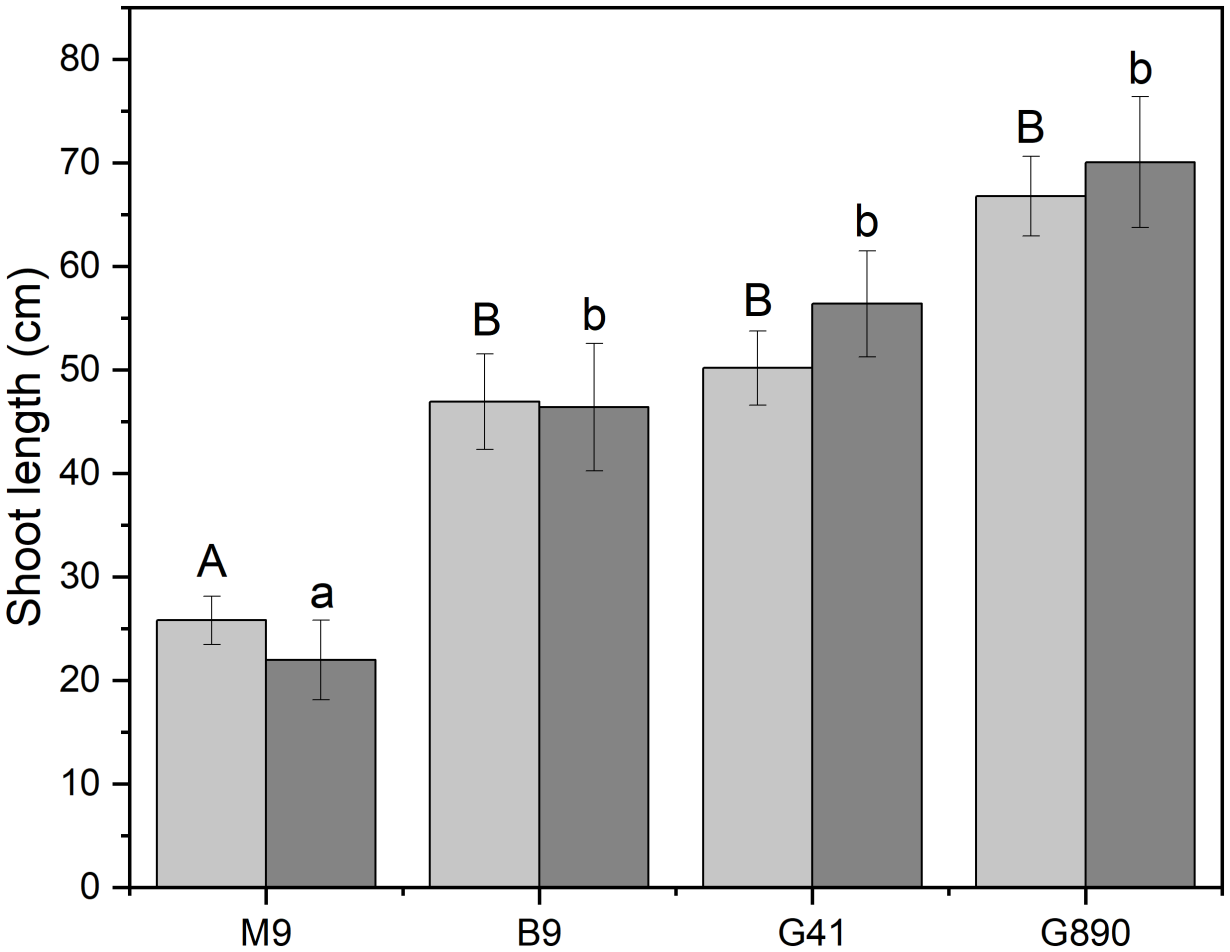
Shoot length for non-grafted trees (light grey) and grafted trees (dark grey) for B.9, M.9, G.41 and G.890 rootstocks. Error bars denote standard error (N=3). Large and small letters indicate significance differences among means for ungrafted and grafted rootstocks, respectively, determined using a Tukey`s HSD mean separation test (α=0.05).

Whether a scion was grafted or left ungrafted, rootstocks strongly mediated shoot length (**Figure 5**). M.9 had the shortest shoot length among all rootstocks with an average shoot length of 25.8 cm for ungrafted trees (P< 0.001). Mean shoot length for ungrafted G.890, G.41 and B.9 were not significantly different. The same rankings for shoot length among rootstocks were also observed in grafted trees. Shoot length of M.9 was the shortest among all rootstocks with an average of 21.9 cm (P<0.01) and G.890, G.41 and B.9 did not differ statistically. However, for both grafted and ungrafted trees, G.890 had more shoot growth compared to the other rootstocks (**Figure 5**).

**Figure 6:**
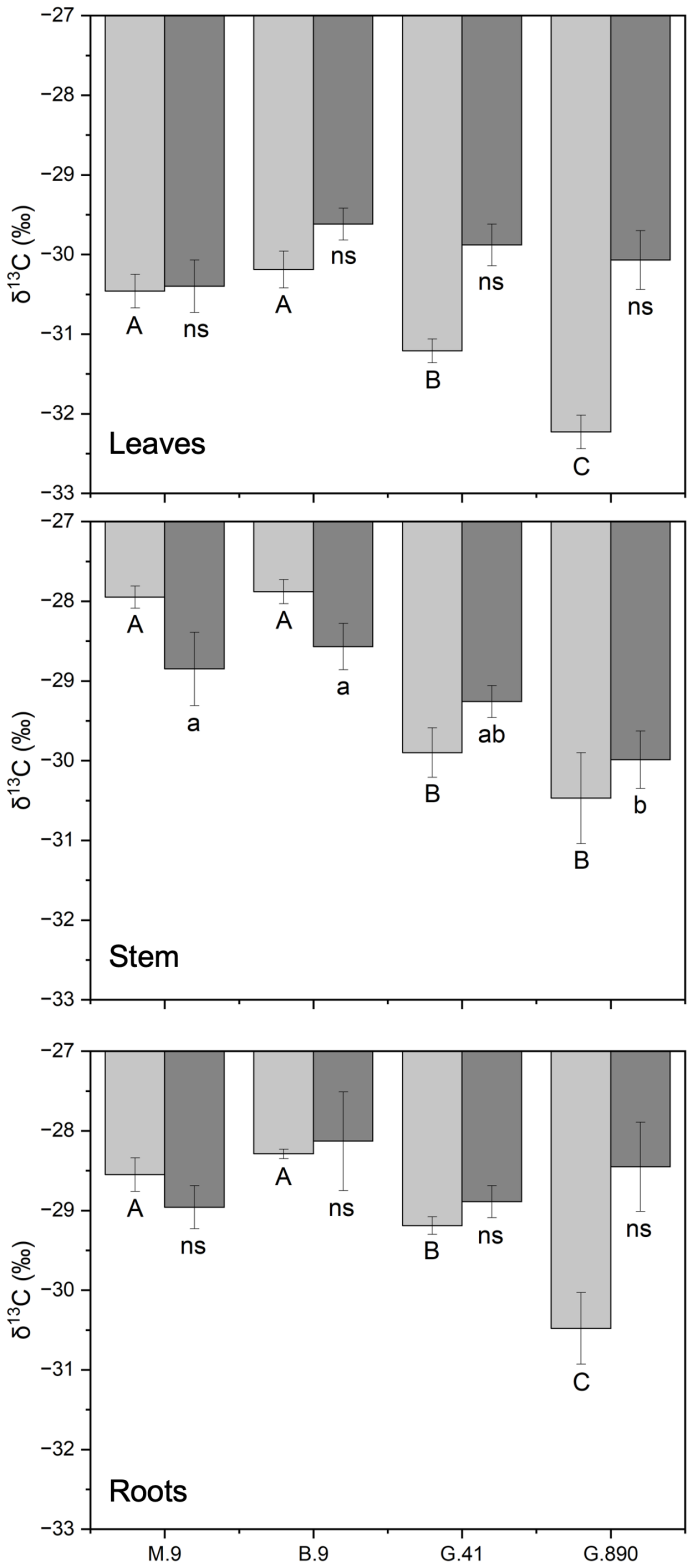
δ^13^C of non-grafted (light grey bars) and grafted trees (dark grey bars) for leaves, stems and roots for B.9, M.9, G.41 and G.890 rootstocks. Error bars denote standard error (N=3). Large and small letters indicate significance differences among means for ungrafted and grafted rootstocks, respectively, determined using a Tukey`s HSD mean separation test (α=0.05). ns indicates that the means are not significantly different.

**Figure 7:**
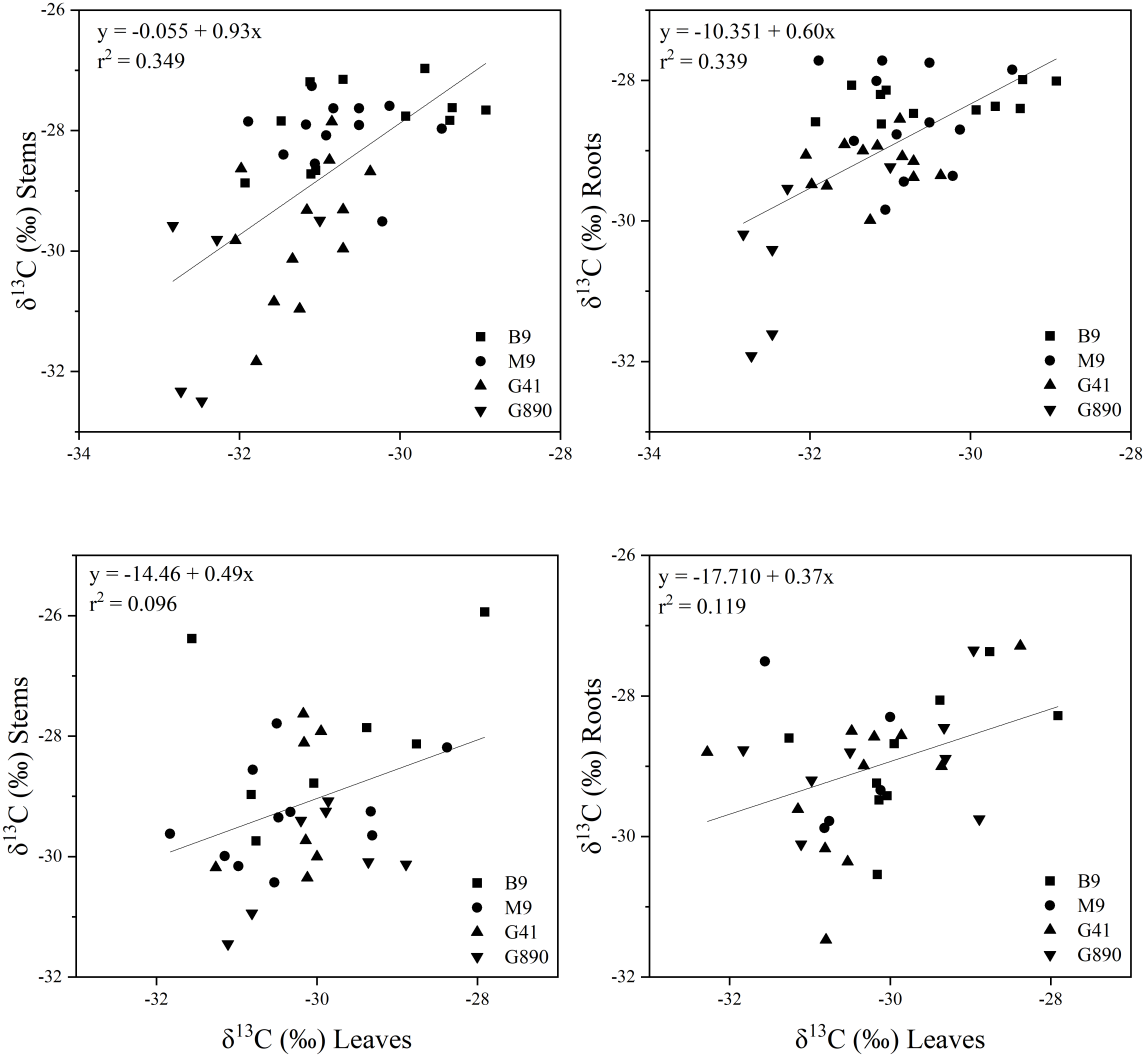
Top graphs: the relationship between δ^13^C leaves and stems (r= 0.605; P<0.001) (Top left) and δ^13^C leaves and roots (r = 0.596; P<0.001) (Top right) for non-grafted trees and between δ^13^C leaves and stems (r = 0.596; P<0.001) (Bottom left) and δ^13^C leaves and roots (r = 0.383; P<0.05) (Bottom right) for grafted trees for B.9, M.9, G.41 and G.890 rootstocks. The lines indicate the best-fit linear relationship for the combined data points.

**Figure 8:**
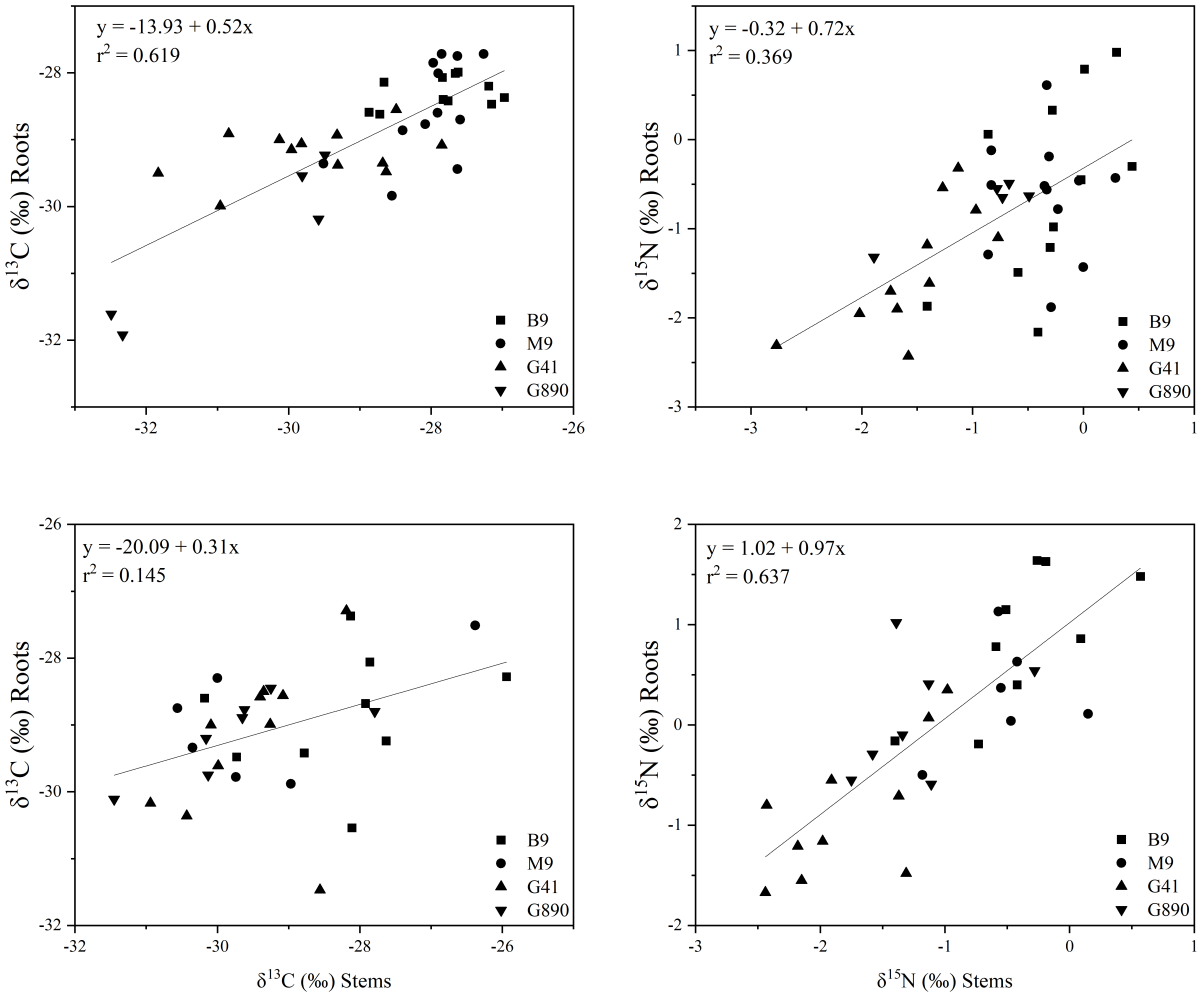
The relationship between δ^13^C stems and roots (r = 0.793; P<0.001) (Top left) and δ^15^N stems and roots (r = 0.613; P<0.001) (Top right) for non-grafted trees. Bottom graphs: relationship between δ^13^C stems and roots (r = 0.416; P<0.05) (Bottom left) and δ^15^N stems and roots (r = 0.416; P<0.05) (Bottom right) for grafted trees for B.9, M.9, G.41 and G.890 rootstocks. The lines indicate the best-fit linear relationship for the combined data points.

Leaf δ^13^C was not different among rootstocks when trees were grafted (**Figure 6**). However, when trees were left ungrafted, mean leaf δ^13^C ranged from -30 to -32 ‰. Leaf δ^13^C was more enriched for M.9 and B.9 and was most depleted for G.890. The same patterns were observed for stems and roots. Stem δ^13^C ranged from -27 to -30 ‰ with G.41 and G.890 more depleted compared to M.9 and B.9. Similarly, root δ^13^C averaged between -28 to -30 ‰ and G.890 and G.41 were more depleted compared to M.9 and B.9. Interestingly, root δ^13^C for grafted trees was not statistically different among rootstocks. However, stem δ^13^C of grafted trees, which belonged to the scion, was like those of ungrafted trees with M.9 and B.9 being more depleted compared to G.890. Moreover, stem δ^13^C was more enriched than leaf δ^13^C for both grafted and ungrafted trees and was strongly affected by rootstocks (**Table 3**). Similarly, the same effect can be observed in the positive correlation between leaf and stem δ^13^C for ungrafted and grafted plants (P<0.001; r = 0.605; P< 0.05, r = 0.353) respectively (**Figure 7**). Overall, grafting appeared to dampen the effects of rootstock on both leaf and stem isotope composition, but not roots. The differences between the rootstocks with the most and least enriched leaf and stem δ^13^C were greater when rootstocks remained ungrafted. Roots δ^13^C was also correlated positively with leaf δ^13^C (P<0.001; r = 0.596; P< 0.05, r = 0.383; **Figure 7**) and stems δ^13^C (P<0.001; r = 0.793; P< 0.05, r = 0.416; **Figure 8**).

**Table 3:** Mean carbon (δ^13^C), oxygen (δ^18^O), and nitrogen (δ^15^N) isotope composition ( ± SEM, N=6) for apple trees on B.9, M.9, G.41 and G.890 rootstocks.

	δ^13^C (‰)			δ^18^O (‰)			δ^15^N (‰)		
	Leaves	Stems	Roots	Leaves	Stems	Roots	Leaves	Stems	Roots
Ungrafted Plants									
M.9	-30.46 ± 0.21a	-27.95 ± 0.14 a	-28.55 ± 0.21 a	17.55 ± 0.26 b	17.55 ± 0.39 a	15.02 ± 0.48 ns	-0.84 ± 0.08 a	-0.36 ± 0.09 ns	-0.63 ± 0.18 a
B.9	-30.19 ± 0.23a	-27.88 ± 0.15 a	-28.29 ± 0.06 a	18.40 ± 0.31 a	16.64 ± 0.28 ab	15.07 ± 0.30 ns	-1.05 ± 0.12 a	-0.29 ± 0.11 ns	-0.57 ± 0.31 ab
G.41	-31.21 ± 0.15b	-29.90 ± 0.31 b	-29.19 ± 0.11 b	18.28 ± 0.24 a	15.88 ± 0.47 bc	15.34 ± 0.41 ns	-1.51 ± 0.13 b	-0.84 ± 0.38 ns	-1.42 ± 0.19 b
G.890	-32.23 ± 0.21c	-30.47 ± 0.57 b	-30.48 ± 0.45 c	17.90 ± 0.20 ab	14.90 ± 0.39 c	14.65 ± 0.41 ns	-0.82 ± 0.14 a	-0.78 ± 0.20 ns	-0.80 ± 0.14 ab
Grafted plants									
M.9	-30.40 ± 0.33 ns	-28.85 ± 0.46a	-28.96 ± 0.27 ns	18.63 ± 0.23 b	15.39 ± 0.52 a	14.69 ± 0.48 b	-0.81 ± 0.23 a	-0.86 ± 0.20 a	0.23 ± 0.26 ab
B.9	-29.62 ± 0.20 ns	-28.57 ± 0.29a	-28.13 ± 0.62 ns	19.82 ± 0.25 a	15.02 ± 0.31 ab	14.11 ± 0.24 ab	-0.97 ± 0.13 a	-0.54 ± 0.12 a	0.82 ± 0.21 a
G.41	-29.88 ± 0.26 ns	-29.26 ± 0.20ab	-28.89 ± 0.20 ns	19.60 ± 0.24 ab	14.63 ± 0.14 ab	13.37 ± 0.31 a	-1.82 ± 0.10 b	-1.52 ± 0.15 b	-0.93 ± 0.20 c
G.890	-30.07 ± 0.37 ns	-29.99 ± 0.36b	-28.45 ± 0.56 ns	19.62 ± 0.29 ab	14.18 ± 0.24 b	14.45 ± 0.30 ab	-1.14 ± 0.15 a	-1.09 ± 0.16 ab	-0.08 ± 0.19 b

Different letters indicate significance differences among means determined using a Tukey`s mean separation test (α=0.05). ns indicates that the means are not significantly different.

**Figure 9:**
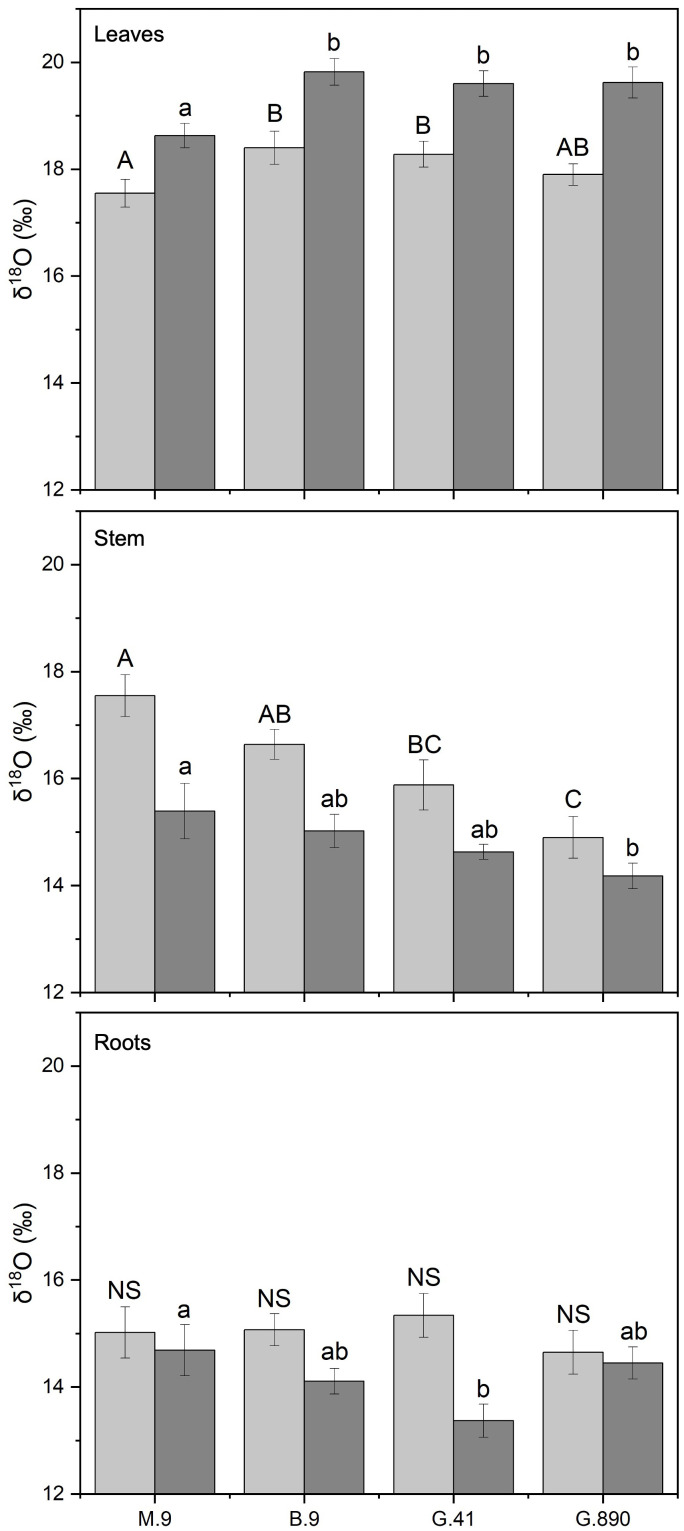
δ^18^O of non-grafted (light grey bars) and grafted trees (dark grey bars) for leaves, stems and roots for B.9, M.9, G.41 and G.890 rootstocks. Error bars denote standard error (N=3). Large and small letters indicate significance differences among means for ungrafted and grafted rootstocks, respectively, determined using a Tukey`s HSD mean separation test (α=0.05). ns indicates that the means are not significantly different.

Leaf biomass δ^18^O was also significantly affected by rootstock. These differences were consistent whether the rootstock was grafted or not. For non-grafted trees, leaf δ^18^O was the lowest for M.9. (**Figure 9**). The same trend was observed for grafted trees with M.9 lower than B.9, G.41, and G.890 (P< 0.05). Differences in stem δ^18^O were also observed among rootstocks. δ^18^O for G.890 was lower compared to M.9, B.9 and G.41 for ungrafted trees (P< 0.001). Similar differences were also observed for grafted trees where G.890 was more depleted compared to B.9 (P = 0.01) (**Figure 9**). Root δ^18^O was not different among rootstocks when trees were grafted (**Figure 9**). However, when trees were left ungrafted, mean root δ^18^O ranged from 13 to 15 ‰ and was highest for M.9 and G.890 (**Figure 9**). For δ^18^O for leaves and stems, leaves were more enriched than stems for both ungrafted and grafted plants, contrary to what was observed for δ^13^C.

**Figure 10:**
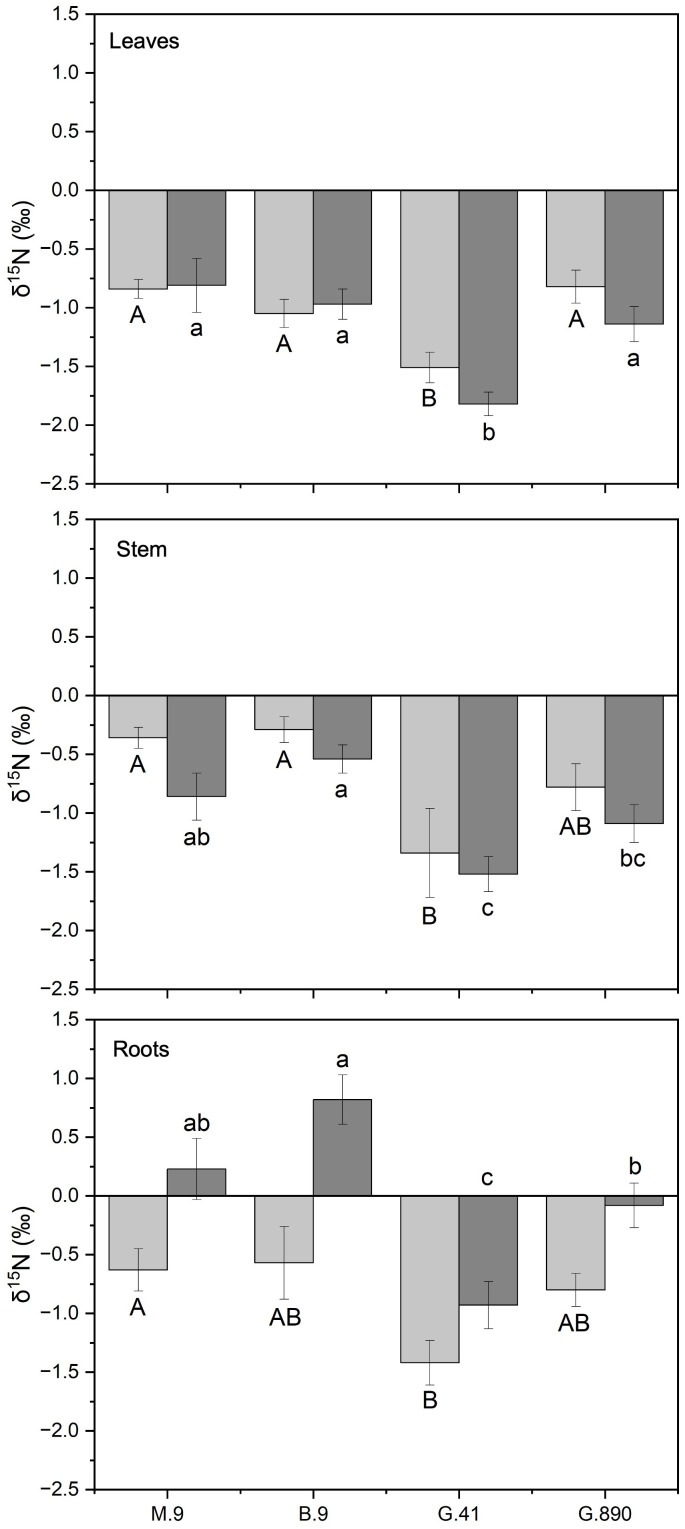
δ^15^N of non-grafted (light grey bars) and grafted trees (dark grey bars) for leaves, stems and roots for B.9, M.9, G.41 and G.890 rootstocks. Error bars denote standard error (N=3). Large and small letters indicate significance differences among means for ungrafted and grafted rootstocks, respectively, determined using a Tukey`s HSD mean separation test (α=0.05). ns indicates that the means are not significantly different.

Differences in δ^15^N were consistently different among rootstocks for both grafted and ungrafted trees. Leaf δ^15^N for G.41 was significantly more depleted compared to G.890, M.9 and B.9 for both grafted (P< 0.001) and ungrafted trees (P<0.001) (**Figure 10**). Leaf δ^15^N for G.41 was approximately 0.8‰ less than other rootstocks. G.41 also showed remarkably negative values compared to M.9, B.9 and G.890, especially for grafted trees (**Figure 10**).

**Figure 11:**
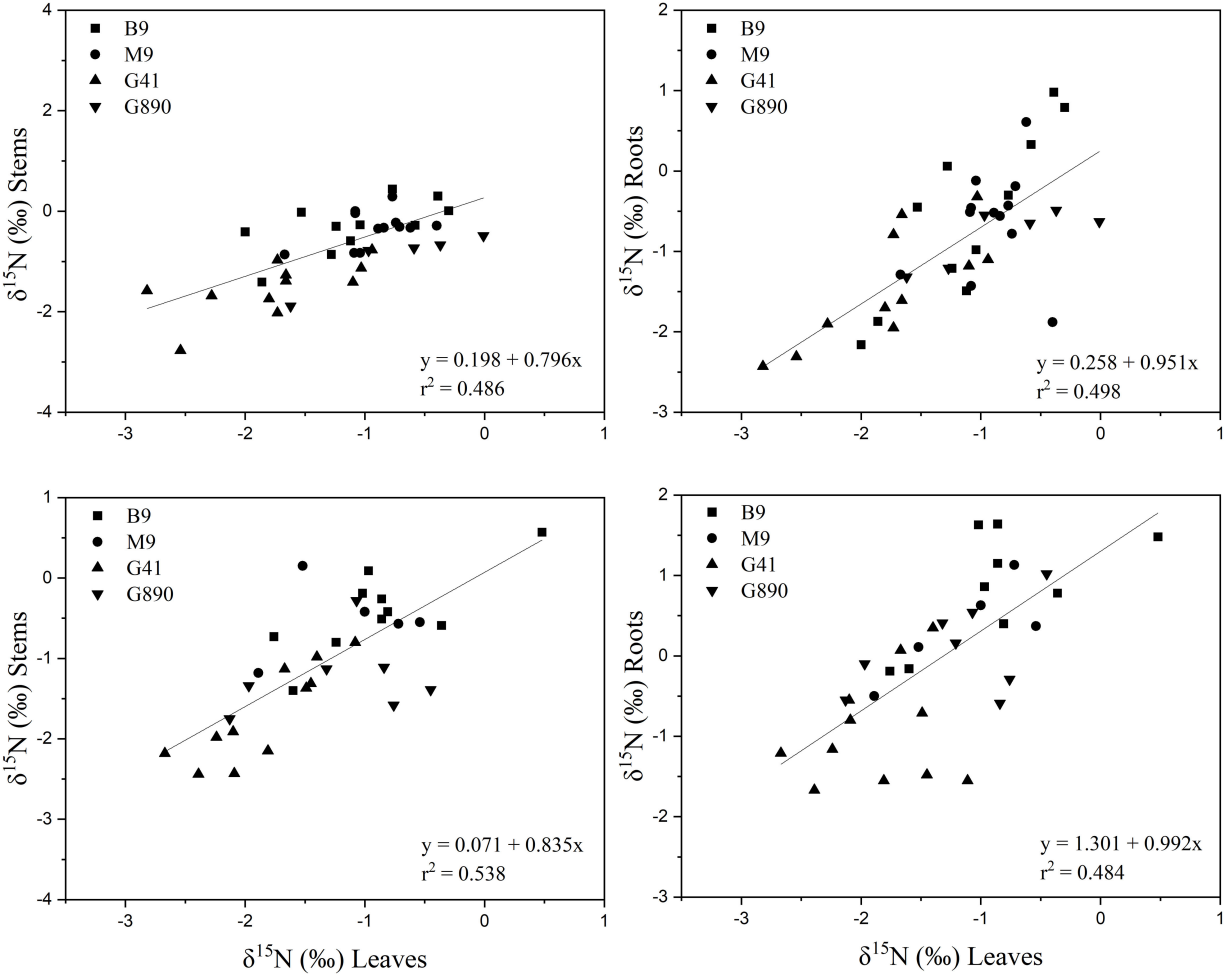
Top graphs: the relationship between δ^15^N leaves and stems (r = 0.552; P<0.001)(left) and δ^15^N leaves and roots (r = 0.713; P<0.001)(right) for non-grafted trees. Bottom graphs: the relationship between δ^13^C leaves and stems (r = 0.743; P<0.001)(left) and δ^13^C leaves and roots (r = 0.707; P<0.001) (right) for grafted trees for B.9, M.9, G.41 and G.890 rootstocks. The lines indicate the best-fit linear relationship for the combined data points.

Leaf δ^15^N correlated positively with stem δ^15^N for ungrafted and grafted plants (P<0.001; r = 0.552; P< 0.001, r = 0.743; **Figure 11**) and with root δ^15^N (P<0.001; r = 0.713; P< 0.001, r = 0.707). There were no patterns of enrichment of stem δ^15^N compared to leaf δ^15^N like there was for δ^13^C. Stem δ^15^N also correlated positively with root δ^15^N for ungrafted and grafted plants (P<0.01; r = 0.442; P< 0.05, r = 0.416) respectively (**Figure 11**).

## Discussion

Here, we report a clear effect of rootstocks on above ground apple growth and physiology under controlled greenhouse conditions. These differences were consistent whether a scion was grafted to the rootstock or the rootstock was left ungrafted. There were strong differences in not only δ^13^C, but also δ^18^O and δ^15^N. Rootstock differences in functional physiology and δ^13^C and δ^18^O corresponded to known differences in vigor among the rootstocks used in these experiments.

G.890 was the most vigorous of the four rootstocks used in this study which is consistent with previous rootstock research (Robinson et al., 2012; Kviklys et al., 2014; Valverdi and Kalcsits, 2021). B.9 was expected to have the lowest vigor (Czynczyk et al., 2000; Xu and Ediger, 2021; Casagrande Biasuz and Kalcsits, 2022). However, M.9 was significantly smaller than the other rootstocks. These results aligned with other studies that reported similar patterns when grafted with ‘Goldspur Delicious’, ‘Redspur Delicious’ progeny (Tworkoski and Miller, 2007), ‘Golden Delicious’, ‘Honeycrisp’, and ‘Fuji’ cultivars (Dallabetta et al, 2021). Slow growth has been reported for M.9 under greenhouse conditions (Valverdi et al., 2019). In this study, root health also appeared to be affected compared to other rootstocks (data not shown). That may have an impact on growth that was inconsistent with dwarfing characteristics observed under field conditions.

Like what was observed previously (Biasuz and Kalcsits, *2022*), δ^13^C was related to shoot vigor. Carbon isotope composition can indicate differences in intrinsic water-use efficiency (Gonçalves et al, 2007; Seibt et al, 2008; Martínez-Alcántara et al, 2013; Foster et al, 2017; Xu and Ediger, 2021). Depleted δ^13^C can indiacate elevated stomatal conductance (Farquhar et al, 1989; Arndt and Wanek, 2002; Liu et al, 2012; Cernusak et al, 2013). However, according to the dual isotope model (Scheidegger et al., 2000), uncoupling of δ^13^C and δ^18^O can indicate that stomatal conductance is not responsible for variations in δ^13^C. Poor agreement between δ^13^C and δ^18^O in leaves suggests that stomatal conductance may not be the key driver of variation in δ^13^C and that another trait imparted by the rootstock itself may have contributed to observed differences in δ^13^C. In support, we observed depleted δ^13^C non-grafted plants for G.890 and enriched values for M.9 and B.9. However, these differences in δ^13^C were not observed for grafted plants with the same scion.

Stem δ^13^C more closely reflected differences among rootstocks in shoot vigor than leaf δ^13^C. Moreover, stems were enriched compared to leaves for δ^13^C. Cernusak et al. (2009) suggested that post-photosynthetic carbon fractionation may be caused by seasonal separation of growth (Cernusak et al., 2009). These patterns of enrichment were also observed for apple under field conditions (Kalcsits et al., 2022). Under field conditions, development and growth of leaves occur in spring with plentiful soil water content. The development and growth of stem tissue occurs later when warmer temperatures and elevated VPD reduce stomatal conductance at leaf level resulting to a less carbon isotope discrimination (Cernusak et al., 2009). Moreover, isotope fractionation can occur in leaves and may change during leaf development. Mature leaves can be the source for carbon used to synthesize heterotrophic tissues like stems which become enriched relative to newly developed leaves on the terminal end of the stem (Cernusak et al., 2009; Werner and Gessler, 2011).

Stem δ^18^O closely followed the same differences observed for δ^13^C. For both grafted and non-grafted plants, we observed depleted values for vigorous G.890 and enriched values for M.9 and B.9 indicating differences in water and carbon assimilation in leaves. Consequently, stem δ^13^C was enriched and δ^18^O was depleted compared to leaves. However, these differences were only observed for higher vigor rootstocks like G.41 and G.890 and were not observed for M.9 and B.9. Not only these effects were consistent for both grafted and ungrafted trees, G.890 had depleted stem δ^13^C and enriched δ^18^O values for both grafted and ungrafted trees, which demonstrates that the impact of rootstock on whole plant water relations can be independent of the scion or the presence of a graft union.

G.41 had significantly more depleted δ^15^N compared to the other two rootstocks and these results were consistent between grafted and ungrafted trees. Evans (2001) and Kalcsits et al. (2014) have described the physiological mechanisms underlying variation in nitrogen isotope discrimination. The δ^15^N (‰) of each source nitrogen used here was -0.73‰, 2.51‰, and 0.03‰ for calcium nitrate, ammonium phosphate, and potassium nitrate, respectively. This experiment was close to an open-source experiment like Kalcsits and Guy (2016) but with smaller nitrogen supply. Therefore, according to proposed nitrogen isotope discrimination models, more depleted leaf and stem δ^15^N could indicate higher efflux from the roots. However, more research will be required to delineate the underlying reasons for different δ^15^N for G.41 compared to the other three rootstocks in this study.

Water limitations imposed by dwarfing rootstocks have been previously reported in woody plants (Bongi et al., 1994; Padgett-Johnson et al., 2000; Gibberd et al., 2001; Cohen and Naor, 2002; Ma et al., 2010; Liu et al., 2012; Galbignani et al., 2016; Peccoux et al., 2018; Villalobos-González et al., 2019; Frioni et al., 2020; Reig et al., 2022). However, close associations between stem water potential and gas exchange were not always observed. Under greenhouse conditions in this study, there were no consistent trends in the association between rootstock-induced vigor and stomatal conductance or stem water potential. The controlled conditions of this study may have dulled previously reported rootstock responses (Casagrande Biasuz and Kalcsits, 2022). Other studies had compared water potential among dwarfing and vigorous rootstocks under different soil conditions. More dwarfing rootstocks generally show more negative stem water potential than the more vigorous rootstocks (Olien and Lakso, 1986). Differences on stem water potential of dwarfing and vigorous rootstocks occur during the day between pre-dawn and midday (Basile et al., 2003). ‘Fuji’ showed lower water potential on M.9 than on MM.111 at pre-dawn, however, under drought conditions both ‘Gala’ and ‘Fuji’ had less negative water potential on M.9 than on MM.111 (Tworkoski and Fazio, 2016). Trees under well-watered conditions showed higher leaf water potential for dwarfing rootstocks and more negative for vigorous rootstocks and at the same time, stomatal conductance was greater for more vigorous rootstocks (Atkinson et al., 2000). Basile et al. (2003) and Giulivo et al. (1984) observed higher water potentials in trees grafted on more dwarfing rootstocks compared to more vigorous rootstocks. Nevertheless, the latter showed higher stomatal conductance associated with higher use of water on trees grafted on vigorous while those on dwarfing were more conservative. Stomatal insensitivity to decreasing leaf water potential provides a means by which growth on vigorous rootstocks is maximized until water supplies are depleted (Atkinson et al., 2000).

Rootstocks can modulate water relations through an impact on the soil-plant-atmosphere continuum. Here, we observed similarities in differences in leaf gas exchange and isotope composition among rootstocks for both grafted and ungrafted plants. Even though graft union may affect the growth and development of plants in some cases, the consistent differences in above ground growth between grafted and non-grafted plants in this study highlighted that roots are a significant contributor to tree related vigor, gas exchange and isotope composition that may be independent of the graft union. Even though Atkinson and Else (2001) observed higher resistance to water flow through the graft union on some dwarfing rootstocks like M.27 compared to the vigorous rootstock MM.106 and M.9 being an intermediate between these two rootstocks, this may not be the case for other studies. Carlson and Oh (1975) reported dwarfing for M.8 rootstocks used as an interstem compared to when M.8 was not used. These reported observations would indicate that physiological limitation in the rootstock itself or the graft union rather than root-based traits contribute to limitations in tree vigor. Cohen et al. (2007) and Adams et al. (2018) both observed a minor resistance to water flow at the graft union. Fresh grafted trees may be more resistant immediately after grafting due to wound formation, but after two months of maturation, trees did not show any sustained hydraulic resistance at the graft union (Gascó et al., 2007). In general, studies on mature trees conclude that the graft union does not play a major role in the dwarfing effect (Nardini et al., 2006; Else et al., 2018).

## Conclusions

Here, both grafted and ungrafted apple trees highlighted the effect of rootstock on above ground growth and gas exchange and its consequential effects on carbon, oxygen, and nitrogen isotope discrimination under controlled hydroponic conditions. This work separated out confounding effects that can sometimes be observed in the field. Although the graft union may affect scion vigor in some cases, rootstock differences in isotope composition were also observed in ungrafted trees indicating that root-based traits are contributing to changes in plant water status and leaf functional physiology. Even under controlled conditions, these effects were associated with rootstock induced shoot vigor and were consistent between grafted and ungrafted trees. These results have implications in understanding the contributions of potential dwarfing mechanisms for apple rootstocks and their effect on gas exchange and water relations with longer term implications for selecting rootstocks that are more tolerant of abiotic stresses.

## Data availability statement

The raw data supporting the conclusions of this article will be made available by the authors, without undue reservation.

## Author contributions

EC: Conceptualization, Data curation, Formal analysis, Investigation, Methodology, Software, Visualization, Writing – original draft, Writing – review & editing. LK: Conceptualization, Data curation, Funding acquisition, Methodology, Project administration, Resources, Supervision, Validation, Visualization, Writing – original draft, Writing – review & editing.
